# 3D-Printed Complete Dentures for Patients With Limited Mandibular Residual Ridges: A Case Report

**DOI:** 10.1155/crid/8849925

**Published:** 2025-09-30

**Authors:** Edgar García Zea, Stephanie Jaramillo, Pablo Lenin Benitez Sellan

**Affiliations:** ^1^Department of Prosthodontics, College of Dentistry and Dental Clinics, The University of Iowa, Iowa City, Iowa, USA; ^2^Independent Researcher, Guayaquil, Ecuador; ^3^Department of Prosthodontics, School of Dentistry, Universidad de Especialidades Espiritu Santo, Samborondón, Guayas, Ecuador

**Keywords:** 3D printing, complete denture, edentulous mouth

## Abstract

Digital workflows have revolutionized complete denture fabrication by enhancing precision, minimizing procedural errors, and improving patient outcomes in terms of fit, stability, and satisfaction. This case report demonstrates the integration of digital and conventional techniques in the rehabilitation of an edentulous patient who presented with severe mandibular ridge resorption. An 87-year-old male with impaired masticatory function and inadequate denture retention was treated using a comprehensive digital protocol that incorporated intraoral scanning, computer-aided design (CAD), and 3D printing technologies. Functional impressions and border molding were performed to capture dynamic oral tissue movements, and CAD software was employed to ensure precise maxillomandibular alignment. A trial denture was digitally designed, tested, and refined before the fabrication of the final prosthesis using 3D-printed materials. Retention was quantitatively evaluated using a digital force gauge, which confirmed significant improvements in the prosthetic performance. This case highlights the efficacy of digital dentistry in overcoming the anatomical challenges associated with edentulous ridges and underscores the need for further research to validate the long-term advantages of digital techniques in prosthodontics.

## 1. Introduction

Mandibular dentures have historically presented significant challenges, primarily because of issues such as insufficient residual ridge height, high tongue mobility, and the presence of movable anatomical structures [1]. These factors often lead to reduced stability and retention of the denture, causing discomfort and functional limitations in patients [2]. To address these challenges, dental professionals have explored various techniques and materials for improving the fit and performance of mandibular dentures [3].

Over time, numerous impression methods have been created to address the challenges associated with dentistry, especially in the area of complete dentures [4]. Among these, Abe's functional techniques, introduced in 1999, have proven effective in creating accurate impressions by focusing on the functional movements of the jaw and mouth, thereby enhancing the precision of dental restorations [5]. This comprehensive approach produces prosthetics with a superior fit and enhanced comfort, functionality, and satisfaction for patients [6].

The advent of digital technology has revolutionized dentistry, particularly in the domain of complete dentures [7]. This technological integration has led to the development of prostheses with durable printed bases, offering substantial advantages over traditional denture materials that are susceptible to dimensional changes due to acrylic shrinkage [8]. As a result, three-dimensional (3D) printed prostheses offer the potential for improved esthetics, biomechanical performance, and overall patient satisfaction, marking a significant step forward in the field of removable prosthodontics [9].

Measuring the retention of complete dentures is a critical aspect of prosthodontics and has significant implications for patient satisfaction and oral function. Previous clinical studies have emphasized the importance of this measurement as it directly correlates with the overall success of denture treatment and patient quality of life [8, 10]. The assessment of denture retention provides valuable information to dental professionals, allowing them to evaluate the effectiveness of their prosthetic designs and make the necessary adjustments [11].

Denture retention and fit assessments involve both objective and subjective methods. Objective methods, such as digital traction dynamometers and 3D scanning technologies, provide quantitative data on the physical properties of dentures [12]. These techniques offer precise measurements of retention force and dimensional accuracy, allowing for standardized comparisons across different denture designs and materials [13].

The purpose of this case report was to present a comprehensive and precise description of the method for accomplishing the mandibular suction technique and to validate the retention of a prosthesis. This report relies on an accurate digital workflow and highlights the benefits of modern digital methods for prosthetic dentistry.

## 2. Case Presentation

**Figure 1:**
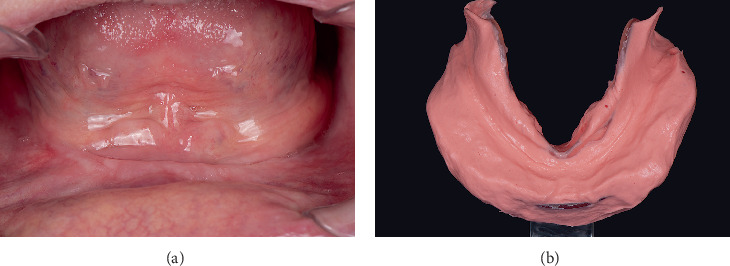
(a) Mandibular residual ridge with insufficient height, illustrating the anatomical challenges affecting denture retention and stability. (b) Primary impression captured using irreversible hydrocolloid, demonstrating detailed reproduction of the patient's oral anatomy.

An 87-year-old Hispanic male presented to a private dental clinic with a chief complaint of impaired masticatory function due to inadequate retention of both maxillary and mandibular dentures. The patient's medical history was noncontributory, with no contraindications for dental treatment. There was no relevant family, genetic, or psychosocial history contributing to the prosthetic condition. He reported using ill-fitting dentures for more than 10 years. Intraoral examination revealed a completely edentulous maxillary arch with a well-formed residual ridge, showing no signs of pathology or factors that could negatively affect prognosis. Examination of the mandibular arch showed a poorly defined retromolar pad, severely resorbed residual ridge, and moderately sized sublingual spongy tissue (Figure 1a). Treatment options for conventional and implant-supported mandibular dentures were discussed, but the patient opted for the conventional approach owing to financial constraints.

The primary impression was taken using a frame cut-back tray with irreversible hydrocolloid impression material (Cavex cream; Cavex, Haarlem, Netherlands) (Figure 1b). This material was selected for its ability to capture accurate anatomical details and provide stable negative reproduction of the patient's maxillary and mandibular arches. Once the primary impression was obtained, it was scanned using an intraoral scanner (Medit i500; Medit, Seoul, South Korea) to create a digital cast. The use of digital scanning enhances precision by eliminating potential distortions that can occur during the conventional pouring of plaster models and provides a reliable foundation for subsequent digital design and fabrication processes.

The extension of the base for the impression was determined to ensure adequate coverage of the functional areas of the arches. A custom tray was designed with precise consideration of anatomical landmarks and functional spaces using dental design software (DentalCAD; exocad GmbH, Darmstadt, Germany). The custom tray was then fabricated using a 3D printing resin (Model V2 Resin; Formlabs, Massachusetts, United States), which ensured a rigid and well-fitting tray that accurately followed the anatomical contours. Once printed, the tray was verified intraorally in the patient's mouth to confirm the proper extension and coverage. The wax occlusion rims were carefully added to the custom printed tray to ensure proper vertical dimension and alignment for optimal patient comfort and function.

During the clinical session, these custom trays with occlusion rims were adjusted intraorally to verify proper occlusal vertical dimension and esthetic parameters, such as the smile line and lip support. Functional movements, including mandibular excursions and phonation, were employed to ensure dynamic adaptation of the prosthesis. Maxillomandibular relationship records were captured using polyvinyl siloxane (bite registration; PlastCare USA, California, United States) and reinforced through digital scanning, allowing precise alignment in the CAD environment.

**Figure 2:**
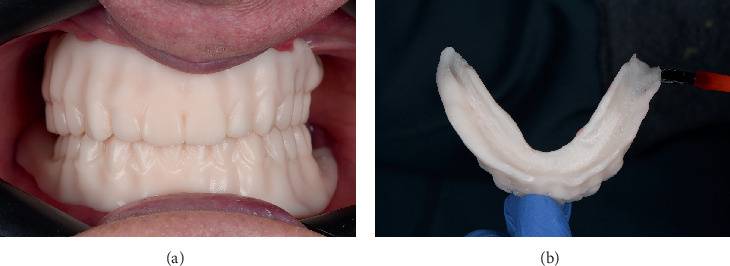
(a) Prototype model designed for denture fitting. (b) Application of adhesive to ensure proper bonding between impression material and the tray surface.

A printed trial denture was designed using a dental design software (DentalCAD; exocad GmbH), incorporating the maxillomandibular relationship records of the patient. During the third clinical session, the fabricated trial dentures (Model V2 Resin; Formlabs, Massachusetts, United States) were positioned in the patient's oral cavity to evaluate tooth alignment, occlusal vertical dimension, and centric relation (Figure 2a). Upon verification of the maxillomandibular relationship discrepancy, the occlusal surfaces of the maxillary posterior dentition were adjusted using acrylic carbide rotary instruments.

**Figure 3:**
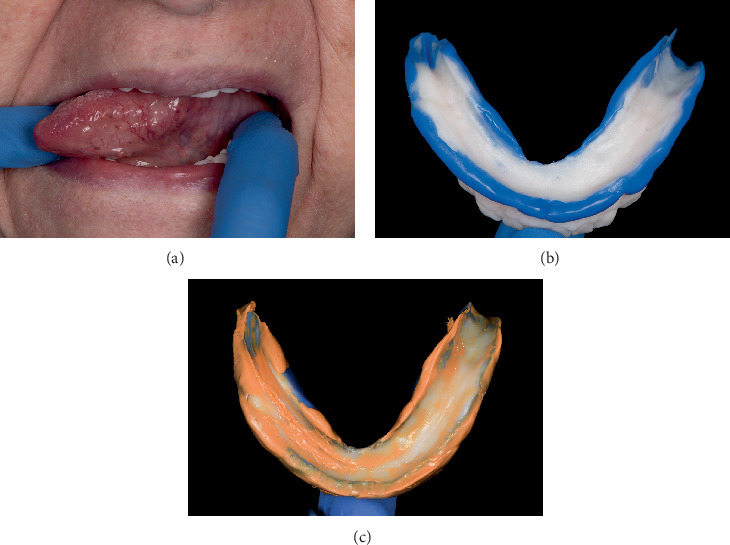
(a) Patient moving the tongue to the right during the functional impression procedure to capture dynamic oral tissue movements. (b) Mandibular border molding procedure using impression material. (c) Final impression capturing precise details of the denture-bearing area after functional movements.

Adhesive (Adhesive Polysiloxane; Coltène, Altstätten, Switzerland) was applied to the tray to enhance the bond between the impression material and the tray surface, and it was allowed to dry completely before continuing with the procedure (Figure 2b). For the border molding procedure, a heavy body vinyl polysiloxane (VPS) impression material (Heavy Body VPS; PlastCare USA, California, United States) was used to capture the dynamic movements of the surrounding oral musculature. The patient was instructed to perform functional movements, such as moving the tongue from side to side (Figure 3a), swallowing, and pushing the tongue toward the incisive papilla. These movements ensured that the border areas of the custom tray captured the full range of functional displacements of soft tissues, leading to a more stable and retentive final prosthesis.

Once the border molding was completed (Figure 3b), the final wash impression was made using a light body VPS (Light Body VPS; PlastCare USA, California, United States) to capture fine details of the denture-bearing areas (Figure 3c). The same functional movements were repeated by the patients during this step to ensure that the final impression accurately reflected the functional state of the oral tissues. The final impression was then scanned digitally to allow for the precise design and fabrication of a digital complete denture.

**Figure 4:**
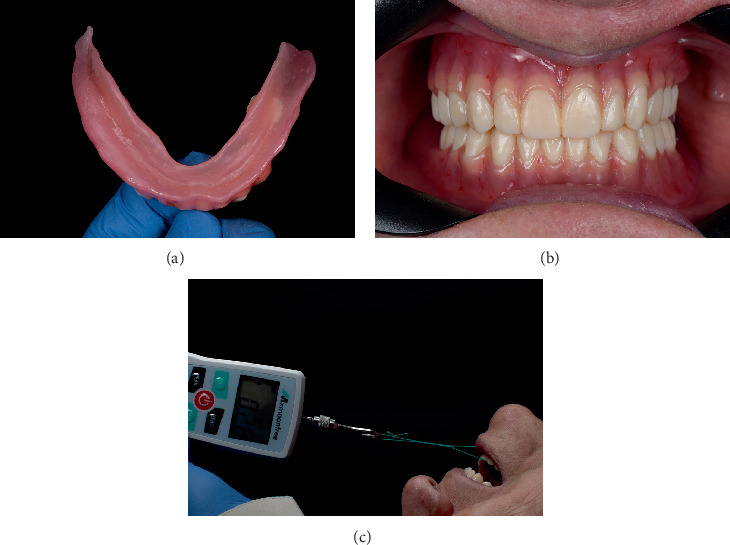
(a) Completed mandibular denture fabricated through 3D printing. (b) Finalized complete denture after all adjustments. (c) Retention testing of the mandibular complete denture using a digital force gauge and dental floss for objective measurement of prosthesis stability.

The digital workflow was continued with the fabrication of a complete denture. The denture bases were printed using a pink base resin (Denture Base LP; Formlabs, Massachusetts, United States) for esthetic and structural properties that mimic the appearance of natural oral tissues (Figure 4a). The denture teeth were fabricated using resin in Shade A2 (Denture Teeth A1; Formlabs, Massachusetts, United States), chosen for their natural appearance and compatibility with the patient's existing dentition (Figure 4b).

The denture teeth are joined to the base by injecting resin into the sockets, pressing the teeth firmly into position, and tack-curing with a light source to secure them. After assembly, the prosthesis undergoes the manufacturer-recommended postcuring cycle to ensure complete polymerization and strength, and the finishing and polishing are performed mechanically using the same rotary instruments and techniques commonly employed in conventional denture protocols.

Finally, the dentures were delivered, and adjustments were made as necessary to optimize fit, comfort, and functionality. Complete denture retention was evaluated using a novel method involving dental floss and a dynamometer (Figure 4c). The prosthesis was tied with dental floss, and the patient was asked to bite down to fully seat the prosthesis. The floss was then attached to the gauge of a dynamometer (ZMF-500N; Mxmoonfree, China) to measure the retention forces and provide an objective assessment of the denture's ability to maintain proper seating under functional conditions. This method offers a reliable and quantifiable measure of denture retention, allowing precise adjustments.

The clinical management followed a structured sequence over several sessions. During the initial appointment, the patient underwent an intraoral examination, and preliminary impressions were taken. In the second visit, digital scanning and custom tray design were performed. The third session was dedicated to border molding, final impression taking, and maxillomandibular relationship registration. The fourth appointment involved the evaluation and adjustment of the printed trial denture. In the final session, the definitive 3D-printed complete denture was delivered. Follow-up visits were scheduled at 24 h, 1 week, and 1 month after delivery. Only at the 24-h appointment was a minor adjustment required, in which a pressure area was selectively relieved using pressure-indicating paste. The patient reported regular use of the prosthesis and no signs of intolerance or discomfort during follow-up appointments. No additional diagnostic testing was required during follow-up, as clinical performance remained satisfactory.

## 3. Discussion

The enhanced dimensional stability of 3D-printed materials offers significant advantages for precision manufacturing and prototyping. This improved stability allows for more accurate reproduction of complex geometries and intricate designs, reducing the need for postprocessing adjustments [14]. Additionally, 3D printing technologies enable the creation of customized parts with consistent dimensions across multiple production runs, leading to increased efficiency and cost-effectiveness in various industries [15, 16].

In contrast, acrylic resins can result in inconsistencies between different production batches. This variability can lead to challenges in maintaining consistent product quality across manufacturing runs [13]. To address this issue, manufacturers often implement strict quality control measures and standardized production processes. However, even with these precautions, slight variations in raw materials or environmental conditions can still impact the final properties of acrylic resin products [3].

Insufficient ridge height and high tongue mobility pose challenges in complete denture fabrication and retention [1]. For ridge height, bone grafting or implant-supported overdentures can improve the prosthesis support and stability. To accommodate tongue mobility, functional impression techniques can be employed to fabricate dentures that harmonize with the surrounding musculature. Additionally, a lingualized occlusion is often selected, as multiple studies have demonstrated that it is a predictable occlusal scheme for complete dentures, offering advantages in patient satisfaction, quality of life, and masticatory performance [17–19]. Denture adhesives and suction cups can provide additional retention. Combining these approaches can optimize functional and esthetic outcomes for patients [20].

Functional impression techniques are essential for accurate oral anatomy capture in complete denture fabrication [6]. This clinical report combined traditional and digital methods to address the challenges posed by the flabby maxillary anterior tissues. A closed-mouth technique recorded functional movements for optimal denture stability and retention, while intraoral scanning captured nonpressurized movable tissues [5]. This integrated approach overcame individual technical limitations, providing a comprehensive and precise record of oral anatomy and function for denture fabrication.

Previous studies have demonstrated that mandibular overdentures with different attachment systems exhibit varying retention forces when measured with a dynamometer [21]. In the present case, a retention force of 1.96 N (≈0.2 kg) was obtained. Although lower than values typically reported for attachment-based systems, this simple approach still provided adequate retention and represents a practical, nonsurgical alternative for completely edentulous patients.

Digital workflows in complete denture fabrication have significantly improved prosthesis fit and retention [8]. This approach uses CAD/CAM technologies, intraoral scanning, and 3D printing to create accurate, personalized dentures. The process captures precise oral anatomy, resulting in better adaptation between the denture base and oral tissues [12]. This improved fit enhances suction and stability, reducing denture displacement. Overall, digital fabrication methods offer superior fit, comfort, and patient satisfaction compared to conventional techniques [22].

The long-term performance of 3D-printed dentures shows promising results when compared to conventional dentures [14]. While further research is needed to fully establish their long-term efficacy, the current findings suggest that 3D-printed dentures may offer a viable and potentially advantageous alternative to traditional denture fabrication methods. As technology continues to advance, it is likely that 3D-printed dentures will play an increasingly important role in prosthodontics, potentially improving patient outcomes and streamlining the manufacturing process.

## 4. Conclusion

The patient reported satisfaction with the comfort and retention of the prosthesis, expressing improved chewing ability and confidence. This case report demonstrates the successful application of digital workflows and 3D printing technology in the fabrication of complete dentures for a patient with limited residual ridges. The integration of traditional impression techniques with advanced digital methods has resulted in a well-fitted prosthesis that addresses the challenges posed by the patient's oral anatomy.

## Data Availability

Data sharing is not applicable to this article as no datasets were generated or analyzed during the current study.
